# Manufacture of low-benzo(a)pyrene sesame seed (*Sesamum indicum* L.) oil using a self-designed apparatus

**DOI:** 10.1371/journal.pone.0173585

**Published:** 2017-03-09

**Authors:** Ji Yoon Yi, Hui Ju Kim, Myong-Soo Chung

**Affiliations:** Department of Food Science and Engineering, Ewha Womans University, Seoul, Republic of Korea; Indian Institute of Chemical Technology, INDIA

## Abstract

The aim of this study was to lower benzo(a)pyrene (BaP) contents in sesame seed oil (SSO) during manufacture by using a self-designed apparatus, to determine its optimal conditions, and to analyze antioxidants in SSO which might be related to BaP content reduction. Washing and spin-drying steps reduce exogenous BaP contamination, and the reduced moisture in seeds lowered BaP content in final SSO. A ventilation system in the roasting step inhibits BaP formation and reabsorption, followed by a controlled compression step. The optimal condition, a single washing cycle with 2-min spin-drying, 1350-rpm ventilation, and a single compression cycle, reduced the BaP content in SSO to 2.93 μg/kg, where the raw seeds had been spiked with 10-μg/kg BaP. Total phenolic contents showed a reversal pattern to the distribution of BaP contents. Sesamol and sesamolin were quantified by a high performance liquid chromatography-ultraviolet detector, and it was suggested that sesamol which is a strong antioxidant might have prevented BaP formation during the roasting step. This study enabled the commercial production of low-BaP SSO, and the data could be used in further investigations of the BaP content reduction mechanism with quantitative chemical analysis of the SSO composition.

## Introduction

**Fig 1:**
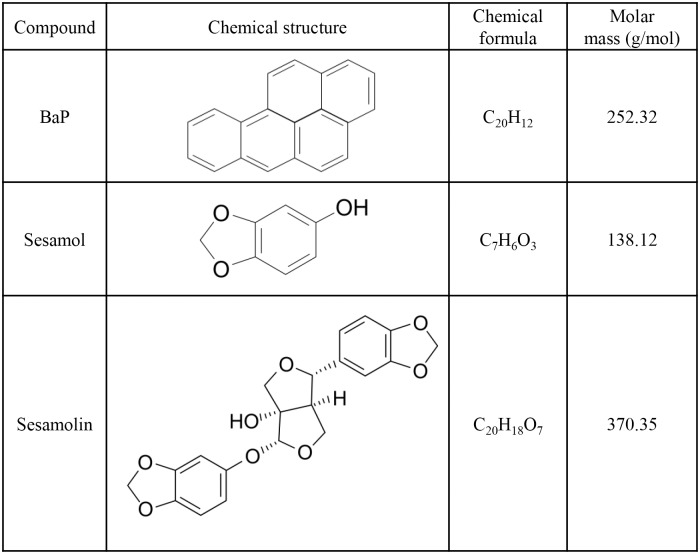
Chemical structures of BaP and major lignans in SSO.

Benzo(a)pyrene (BaP) is a carcinogenic polycyclic aromatic hydrocarbon (PAH) found in various foodstuffs, such as meats, seafoods, dairy products, cereals, grains, fruits, vegetables, breads, and beverages (Fig 1) [1–3]. It was reclassified as an IARC Group 1 carcinogen in 2012, which means that it is carcinogenic to humans [4]. The European Food Safety Authority and the U.S. Department of Health and Human Services have also stressed the potential risks of BaP, since it has been reported to produce tumors at various sites in different species [5,6]. PAHs including BaP are generally produced by the incomplete combustion of organic matter [7] and the pyrolysis of nutrients in foods [3,8]. It has been reported that food processing methods involving thermal treatments such as smoking, roasting, grilling, toasting, and broiling result in the formation of BaP [3,8,9]. Since many vegetable oils are produced using thermal processes and are lipophilic, BaP is also found in various types of vegetable oils [10–12].

Sesame seed (*Sesamum indicum* L.) oil (SSO) is widely consumed as a healthy cooking or seasoning oil in Asia [13], but concerns have been raised regarding its content of BaP, which is mainly formed during the extraction of SSO from sesame seeds (SS) through roasting and compression processes under high temperature. SS are highly nutritious thanks to their high contents of fat (42–54%) and protein (22–25%) [14]. Also, they are abundant in the unique antioxidant substances sesamol, sesamin, and sesamolin, which are classified as lignans. Especially, sesamol and sesamolin have higher oxidative stability compared to other antioxidants in edible oils [15]. These properties contribute to the long shelf life of SSO in addition to its characteristic flavor [16]. However, BaP can be formed during the production of SSO since this involves thermal roasting and compression processes. As a result, The European Commission (2006) [17] and the Korean Ministry of Food and Drug Safety (KMFDS) (2015) [18] have adopted legal limits of 2 μg/kg for BaP in edible oils and fats.

BaP in SSO generally originates from either contamination of raw SS by environmental sources or formation during thermal processing. The routes of PAH contamination in vegetable oils include uptake by oil plants from soils, water, and atmospheric pollution [1,8,19]. It has also been reported that the use of combustion gases to dry seeds [19] and the storing of seeds in jute bags treated with organic matter [12] may result in contamination by PAHs. When SS are roasted, the pyrolysis of their abundant fats and proteins reportedly results in BaP formation, which is a major cause of BaP in SSO [2,10,20,21]. Previous studies have found that the probable mechanisms for BaP production during thermal processing involve molecular reactions of nutrients, such as free radical reactions, intramolecular cyclization, and polymerization [22,23]. There have also been several studies investigating methods for reducing the content of BaP in edible oils via refining methods such as neutralizing, bleaching, and deodorizing the final oil products [24,25]. Furthermore, several studies have reported the effects of lignans in SSO on inactivating BaP [26,27]. However, BaP content reduction during the manufacturing process has not been reported previously.

The aim of this study was to manufacture low-BaP SSO using a self-designed apparatus in our laboratory, to determine the optimal processing conditions, and to examine antioxidants in SSO that are related to reducing BaP content. The invented apparatus employs a washing process to reduce the BaP originating from environmental contamination, followed by a self-designed spin-drying process, which was implemented to study the moisture effects of SS before roasting on the reduction in BaP contents in SSO. A ventilation system is included to prevent BaP formation and its reabsorption into SSO, and the effect of compression conditions was also addressed. By using different processing conditions, significant decrease of BaP content in SSO was achieved. The acid and iodine values of manufactured samples were measured to check for variations in oxidative stabilities, and total phenolic contents were measured to further discuss antioxidative capacities. Finally, sesamol and sesamolin contents were quantified by a high performance liquid chromatography-ultraviolet detector (HPLC-UV) to suggest a probable mechanism for reduction of BaP contents.

## Materials and methods

### Materials and chemicals

The SS used in this study were acquired from a local market (Yoo Lim Oil Manufacture, Seoul, Korea), which were collected and imported from China during 2014 and 2015. They were stored in sacks before usage at a room temperature of 24 ± 2°C (mean ± range). HPLC-grade acetonitrile, *n*-hexane, water, and methanol were purchased from J.T. Baker (Phillipsburg, NJ). BaP standard was from WAKO (Osaka, Japan). Ethanol, ethyl ether, potassium hydroxide, chloroform, iodine, acetic acid, potassium iodide, sodium thiosulfate, starch indicator, and sodium carbonate, were from DUKSAN (Ansan, Korea). Thymolphthalein was from DAEJUNG (Siheung, Korea). Bromine was from SAMCHUN (Pyeongtaek, Korea). Folin-Ciocalteu reagent, and gallic acid standard were from Sigma-Aldrich (St. Louis, MO). Sesamol standard was from Chengdu Biopurify Phytochemicals Ltd (Chengdu, China). Sesamolin standard was from ChemFaces (Wuhan, China).

### The self-designed apparatus

**Fig 2:**
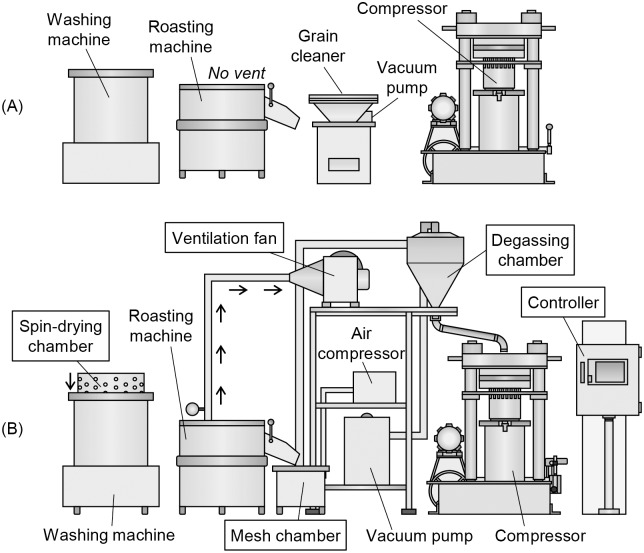
Schematic diagrams of two sets of SSO manufacturing apparatus. (A) Traditional apparatus. (B) The self-designed apparatus for manufacturing low BaP SSO. Changes are highlighted in square boxes.

The apparatus developed in our laboratory for manufacturing low-BaP SSO comprises the following subdevices: a system controller, a washing machine, a roasting machine with a ventilation system, a mesh chamber and a degassing chamber for delivering intermediates, and a compressor (Fig 2). Unlike conventional SSO manufacturing devices, the invented apparatus includes an automatic control system that allows all of the experimental conditions to be maintained automatically at preset values. The system can also monitor the processing conditions of each device in real time.

#### Washing machine

The washing machine in the invented apparatus differs from conventional ones in that it has an automatic controlling method and a spin-drying process for removing moisture of SS before roasting to reduce the BaP content. After pouring raw SS into the washing machine, pressing a start button for the washing process in the system controller, opens the inlet valve and allows fresh water to flow into the machine. When the water level reaches the preset height, the SS are washed by slowly rotating the chamber inside the machine at a rate of 750 rpm, with alternating clockwise and counterclockwise vortices. The outlet of the machine is opened when a 2-min single cycle of washing is completed, which drains the used water. The spin-drying process starts when all of the water has drained out. The chamber containing washed seeds rotates at the high speed of 3,600 rpm, which removes moisture remaining in the seeds by centrifugal force. This continues for the preset duration of spin-drying.

#### Roasting machine

The unique feature of the invented roasting machine is the addition of the ventilation system. Roasting is implemented by pouring SS that have been washed in the washing machine into the roasting machine, which is preheated to 250°C. The roasting machine is pot-shaped, and has an electrical heater attached to its bottom part. When the start button for the roasting process is pressed, a rotating knife stirs the seeds in order to keep them from burning and ensuring a uniform heat distribution. The ventilation system is directly attached to the roasting pot to promote the circulation of roasting gases, which facilitates the removal of gases that include BaP. An air vent attached to the other side of the pot helps this process by supplying fresh new air into the pot. The ventilation rate is controlled by the frequency of the power supply to a ventilation fan (DB-200, DongKun Industrial, Incheon, Korea), and can be set from 0 to 60 Hz, which corresponds to rotation speeds from 0 to 1,800 rpm (Eq 1)).

$$N_{s}=120\times f/p$$ (1)

where *N*_S_ is the synchronous speed (rpm), *f* is the frequency (Hz), and *p* is the number of poles (the fan used in this study has four poles). In addition to assigning preset values for the heating temperature and ventilation rate, the system controller is also able to monitor the temperature of the seeds, which is used to ensure a uniform finishing point for the roasting step. Generally, an appropriate degree of roasting of SS is achieved when 6 kg of them reach 215°C.

#### Mesh and degassing chamber

Roasted seeds are delivered directly into the compressor via both mesh and degassing chambers, which change them into the appropriate state for compression. Pushing down the handle attached to the outlet of the roasting machine results in roasted seeds falling into the mesh chamber. This is used to eliminate large particles, usually comprising foreign materials such as soil contamination, or aggregates of burned substances. When the vacuum pump (NWR040S, Nawoo Machinery, Gimpo, Korea) is activated by pressing a start button for the delivery process, SS in the mesh chamber are transferred to the degassing chamber, and this process is coupled with the vibrating action of the air compressor (LD1501, Geunpoong Power Tool, Seoul, Korea). The degassing chamber removes gases containing BaP along with some small particulates by vacuum force. The circulation of air in this process can also cool the seeds, and so it is important to control the temperature before the compression step.

#### Compressor

Sensors were added to the compressor for monitoring the processing temperature and pressure in order to minimize the formation of additional BaP. After the seeds have been transferred into the compressor, a 6-kg weight is manually placed on top of them to exert pressure, and the compression step starts after closing the lid and pressing a start button. A high pressure of 58.8 MPa is applied to the seeds in order to destroy the cell walls and ensure the release of oils. The overall temperature needs to be maintained at 150°C, and this is controlled by the automatic system.

### Optimization of BaP lowering effect

The standard processing conditions were varied in different ways in order to evaluate their effects on the BaP content in SSO. The standard processing conditions for 6 kg of raw SS were based on modifications to traditional methods as follows: a single washing cycle, 2 min of spin-drying, a ventilation rate of 1350 rpm, and a single cycle of compression. The effects of applying no, one, two, and three washing cycles and 0, 1, 2, and 3 min of spin-drying on the BaP lowering effects were determined By using a conventional mechanical convective oven (KCO-150, Kukje Engineering, Goyang, Korea), additional thermal drying periods of 0, 3, 5, and 10 min were applied to examine the relationship between the moisture content of SS before roasting and the BaP content in SSO extracted from them. The effects of ventilation rates of 0, 450, 900, 1350, and 1800 rpm on the reduction of the BaP content were also determined. Since compression needs to be performed at least once to produce SSO, one, two, and three cycles of the compression process were applied to investigate the effect of repeated compression on the BaP lowering effect in SSO. All experiments were conducted in triplicate, and SSO samples were stored in the dark at a room temperature before being analyzed.

### BaP analysis

The initial raw SS were spiked with 10-μg/kg BaP solution so that the BaP lowering effects in SSO could be assessed. BaP standard was dissolved in acetonitrile to obtain a 100-μg/kg BaP solution. A 0.6-l aliquot of the solution was uniformly sprayed into 6 kg of raw SS with stirring, and the sample was dried with cool air using a portable dryer.

The final oil samples were analyzed by the GC/MS method from the Bio-Medicinal Technology Laboratory at Korea University (Sejong, Korea) within 1 month after performing the compression, since BaP is reportedly stable within this time period [28]. All analyses were carried out at room temperature and in triplicate. It was verified in the preliminary experiment that BaP-spiked SS showed a BaP content of 10 μg/kg after spiking, and the invented apparatus did not introduce any additional contamination by BaP.

### Determination of acid and iodine value

The acid and iodine values of SSO were measured according to the methods described in the Korean Food Code (KMFDS, 2015) [29]. The acid value is the amount of KOH in milligrams required to neutralize 1 g of test sample, and it quantifies the amount of free fatty acids. Ten grams of SSO was dissolved in 100 ml of ethanol-ethyl ether 1:2 (vol/vol), and titrated with 0.1-N KOH using 1% thymolphthalein solution as an indicator. The iodine value is the amount of iodine in grams consumed by 100 g of test sample and was determined using the Hanus method. Ten milliliters of CHCl_3_ and 25 ml of Hanus solution were added to 0.2 g of SSO, and the mixture of solution was stored in the dark for 1 h. Thirty milliliters of 1-N KI and 100 ml of distilled water were then added with stirring so to titrate the solution with 0.1-N Na_2_S_2_O_3_ using starch indicator. The samples were analyzed within 2 months of storage. All analyses were conducted at room temperature and in triplicate.

### Determination of total phenolic content

Total phenolic content of SSO was determined according to the Folin–Ciocalteu micro-method [30,31]. Compounds that have antioxidative capacity other than phenolic compounds are also measured by this method. A 20 μl of SSO was mixed with 1.16 ml of distilled water and 100 μl of Folin–Ciocalteu reagent, followed by the addition of 300 μl of 20% Na_2_CO_3_ solution. Using a shaking incubator maintained at 40°C, the mixture was incubated for 30 min and its absorbance was measured at 760 nm. The phenolic content was then expressed as gallic acid equivalent using Eq 2 based on the standard calibration curve of gallic acid.

$$A=0.98C+1.19\times 10^{-1}(R^{2}=0.99)$$ (2)

where *A* is the absorbance and *C* is the concentration expressed as gallic acid equivalent in SSO (mg/g). All analyses were conducted at room temperature and in triplicate.

### Identification of lignans by HPLC-UV analysis

The lignans sesamol and sesamolin in SSO were analyzed using a HPLC-UV (1260 series, Agilent Technologies, Waldbronn, Germany) with a Zorbax Eclipse Plus reverse phase C8 HPLC Column (5μm, 250 mm × 4.6 mm, Agilent), according to the modified method of Lee et al. [13] and Shin et al. [32] For sesamol analysis, a SSO sample was dissolved in *n*-hexane to prepare a 0.1-g/ml solution and then filtered through a 0.45-μm PVDF membrane (Whatman, Maidstone, UK). The mobile phase was composed of distilled water and acetonitrile with a ratio of 35:65 v/v at a flow rate of 1.0 ml/min. An 80 μl of sample aliquot was injected and sesamol was detected at 290 nm. The standard curve was obtained using a linear regression (*R*^2^ = 0.9975) for sesamol at 5–200 mg/l. For sesamolin analysis, a SSO sample was diluted in 70% methanol (0.2 g/ml) and vortexed (KMC-1300V, Vision Scientific, Seoul, Korea) for 3 min, followed by centrifugation (406g, GTROZEN, Seoul, Korea) for 15 min at 1912 × g. After removing the supernatant, the residue was used for re-extraction. The extract was then filtered through the same PVDF membrane prior to HPLC-UV anlaysis. The mobile phase was 70% methanol at a flow rate of 1 ml/min. A 10 μl of sample aliquot was injected and sesamolin was detected at 290 nm. The standard curve was obtained using a linear regression (*R*^2^ = 0.9937) for sesamolin at 50–1000 mg/l.

All analyses were conducted at room temperature and in triplicate.

### Statistical analysis

The data are indicated as means with their standard deviations. The SPSS Statistics software (version 22, IBM SPSS, Chicago, IL) was used for the statistical analysis. A one-way analysis of variance was used to determine differences among the values with a probability cutoff of *p* < 0.05, with Tukey’s test for BaP contents, acid values, iodine values, and total phenolic contents, and Duncan’s test for sesamol and seamolin contents. Spearman’s correlation coefficients were also calculated with a probability cutoff for statistical significance of *p* < 0.05.

## Results and discussion

### Washing cycle and BaP content reduction

**Fig 3:**
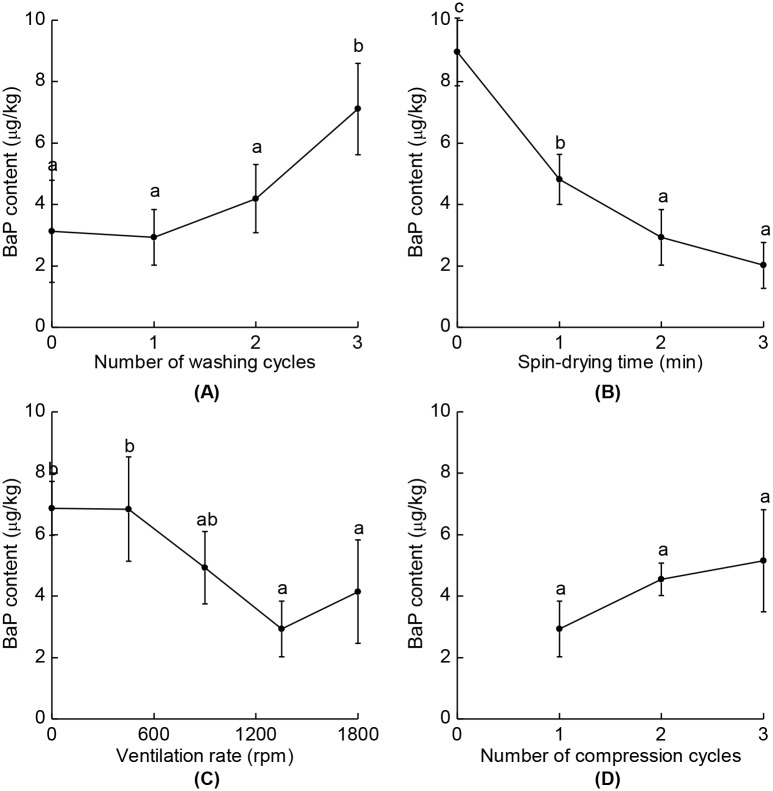
BaP contents in SSO for different processing conditions. (A) Numbers of washing cycles, (B) spin-drying times, (C) ventilation rates, and (D) numbers of compression cycles were varied. The raw SS were initially spiked with BaP at 10 μg/kg. Different letters indicate significant differences between the values (*p* < 0.05). The data are means with their standard deviations (*n* = 3).

The washing step is necessary to eliminate dirt, dust, and some BaP attached to raw SS, while poorly controlled processing conditions lead to an elevated BaP content in SSO, which is produced during the roasting step due to the increased moisture content of SS. Applying a washing cycle to raw SS will remove dirt and dust from them when the water used for washing is drained out. This was verified by the turbidity of the drained water, which became more transparent after each repeated cycle. Fig 3A illustrates the BaP lowering effects on SSO for different numbers of washing cycles. We assumed that the majority of the loosely attached BaP should be eliminated from the washing step. However, the BaP content in SSO extracted from unwashed SS was reduced to 3.13 μg/kg, and since we only omitted the washing here but used the standard conditions for the other factors, a processing step other than washing might have eliminated the majority of the spiked BaP. Indeed, the 1350 rpm of ventilation used for this condition is shown to have strong BaP content reducing effect (see Ventilation Rate and BaP Content Reduction). The application of one or two cycles of washing process did not produce any significant differences in the reduction effects on BaP in SSO compared to unwashed ones: the BaP contents were reduced to 3.13, 2.93, and 4.79 μg/kg in SSO for no, one, and two cycles of washing, respectively. However, it was indicated that increasing the number of washing cycles actually increased the BaP content in SSO, as the application of three washing cycles resulted in 7.11 μg/kg of the BaP content in SSO. Repeated washing cycles also resulted in more moisture absorption by SS (data not shown), and this phenomenon generally induces dehulling of the seeds [33]. Johnson, Suleiman, & Lusas [34] reported that the protein and fat contents of sesame meal are increased when it is dehulled, since the hull is mainly composed of fiber. Since the protein and fat contents contribute greatly to BaP formation during the roasting step, [10,20] SSO extracted by roasting dehulled SS consequently would show a significantly higher BaP content. Moreover, it has been reported that dehulled SS was more abundant in glutamic acid than other amino acids, [34] which is one of the causes of PAHs formation [21]. Thus, in terms of the time and energy efficiencies, the optimal processing condition for the washing process appears to be a single cycle.

### BaP content reduction by lowering moisture of SS

We hypothesized that the washing step would physically remove some of the BaP in raw SS even though moisture uptake results in the formation of additional BaP during the roasting step, and so drying steps were added to control the moisture content after the washing step, resulting in further reductions in the BaP content in SSO. The reductions for different times of spin-drying processing are shown in Fig 3B. Spin-drying for 1 min reduced the BaP content in SSO to 4.81 μg/kg, compared to 8.98 μg/kg when the spin-drying step was omitted. Also, 2 and 3 min of spin-drying showed larger reductions, resulting in 2.93 and 2.02 μg/kg of the BaP contents, respectively. This difference was not significant difference, and so it can be concluded that 2 min of spin-drying is sufficient to reduce the moisture content of washed SS before applying the roasting step.

**Fig 4:**
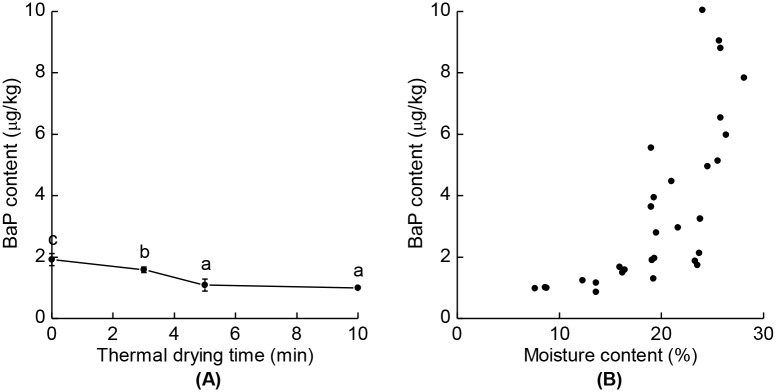
Relationship between the BaP contents in SSO and the moisture content of SS. (A) The BaP contents in SSO for different thermal drying times. (B) Correlation between the moisture content of SS before roasting and the BaP contents in SSO. The raw SS were initially spiked with BaP at 10 μg/kg. Different letters indicate significant differences between the values (*p* < 0.05). The data are means with their standard deviations (*n* = 3). The Spearman’s correlation coefficient was 0.832 (*p* < 0.05, *n* = 3).

Since spin-drying was relatively inefficient at removing the moisture (the moisture content changed from 25.9% to 19.3% after 2 min of spin-drying), a conventional thermal drying step was temporarily implemented to clarify the linear relationship between the moisture content of SS before roasting and the BaP content in SSO extracted from them. The reduction effects of the thermal drying step are shown in Fig 4A. Thermal drying for 0, 3, 5, and 10 min reduced the BaP contents in SSO to 1.98, 1.58, 1.09, and 0.99 μg/kg, respectively; the corresponding moisture contents in SS after the drying processes were 23.5%, 16.2%, 13.2%, and 8.3%, respectively. The Spearman’s correlation coefficient for the correlation between the moisture content of SS before roasting and the BaP content in SSO was 0.831, (Fig 4B). Although this appears to indicate the presence of a strong linear relationship between these two factors, the relationship actually appeared to be exponential or polynomial, and so further investigations are required to examine the exact relationship between them.

### Ventilation rate and BaP content reduction

The addition of a ventilation system to the roasting machine successfully reduced the BaP content in SSO. The reductions in the contents of BaP in SSO are shown in Fig 3C. Ventilation at rates of 450–900 rpm did not exert a significant effect on BaP content reduction compared to the SSO manufactured without the ventilation. However, 1350-rpm ventilation produced a statistically significant decrease in BaP content, which resulted in 2.93 μg/kg of the BaP content in SSO. Also, since the standard processing condition involved ventilation at 1350 rpm, it can be explained that most of the BaP content reduction in an unwashed condition was attributable to the ventilation system, indicating that ventilation is the key factor in reducing BaP in this study. Gfrerer & Lankmayr [11] reported that roasting at high temperatures resulted in damage to the cell walls of seeds and denaturing of the proteins, causing oils to be released from them and then absorb some lipophilic compounds. Since the gases generated from the roasting of fats would contain PAHs [35], and oils are major contributors to the PAHs contamination of foodstuffs due to their lipophilic nature [12,36], not only SSO but also roasted SS are likely to be contaminated by BaP. The ventilation system reduced the contents of BaP in SSO by eliminating such gases, and this could prevent the reabsorption of BaP into roasted SS or SSO. Moreover, ensuring a continuous supply of fresh air by ventilation was reported to significantly reduce the BaP content [22]. However, a further increase in the ventilation rate does not necessarily improve the reduction ability. This could be attributed to the increased ventilation resulting in a nonuniform heat distribution during roasting; the longer roasting time required to reach the desired temperature of SS could result in some of the seeds close to the heating source being exposed to excessive heat [2,10,20]. Therefore, in terms of the BaP reduzction efficacy, ventilation at 1350 rpm was an optimal condition for the roasting process.

### Compression cycle and BaP content reduction

Repeating the compression did not significantly affect the reduction in BaP contents in SSO, despite the compression step involves a high processing temperature and high pressure. The reduction effects for different numbers of compression cycles on BaP in SSO are shown in Fig 3D. Since the compression step involved a high pressure of 58.8 MPa, we supposed that the compression process would further destroy the fibers in roasted SS, resulting in additional BaP production with maintained heat from both the roasting and compression steps. The means of the BaP contents in SSO showed slight decreases when the number of compression cycles was increased, but the differences were not statistically significant regarding their standard deviations. In contrast to the conventional compressor, the invented apparatus includes sensors for monitoring both the operating temperature and pressure, which enables automatic control of the processing conditions in the compression step. This prevents excessive heat occurring when using the manual heater with repeated compression cycles, thereby avoiding the formation of additional BaP. Since repeating the compression cycles was also ineffective at improving the oil yield, a single compression step would be an optimal processing condition.

### Acid and iodine value

**Fig 5:**
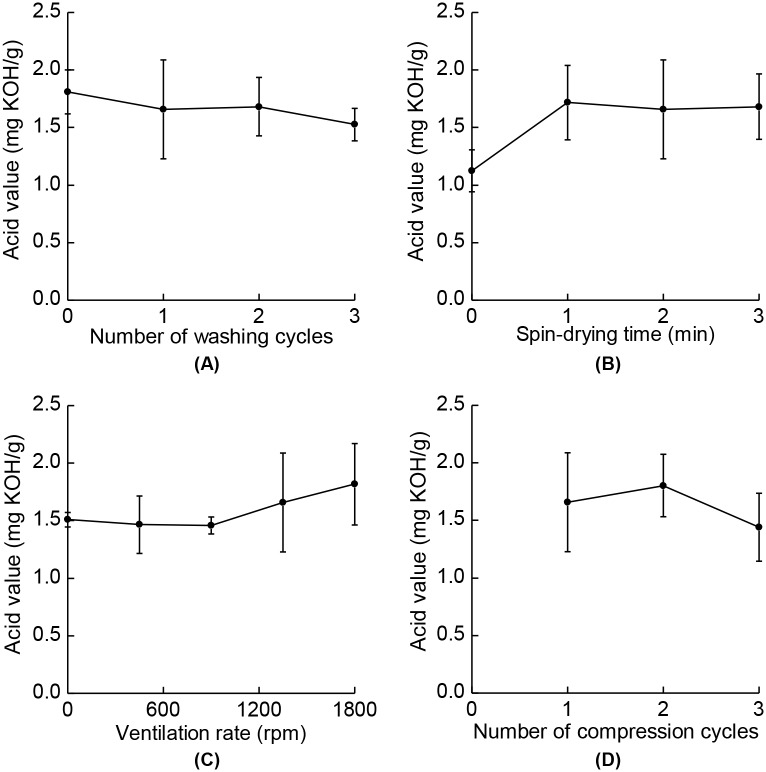
Acid values of SSO for different processing conditions. (A) Numbers of washing cycles, (B) spin-drying times, (C) ventilation rates, and (D) numbers of compression cycles were varied. There were no significant differences between the values (*p* > 0.05). The data are means with their standard deviations (*n* = 3).

**Fig 6:**
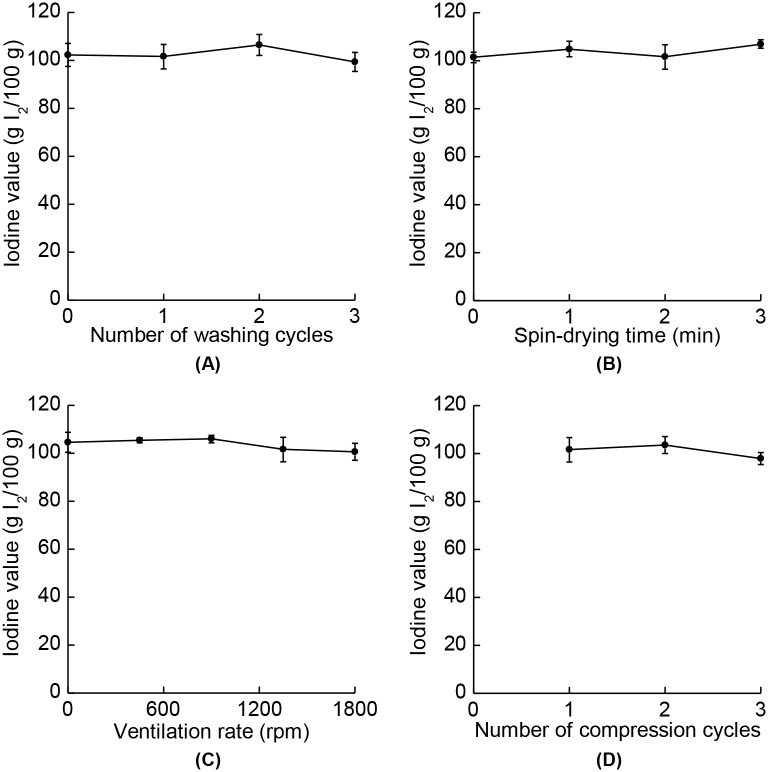
Iodine values of SSO for different processing conditions. (A) Numbers of washing cycles, (B) spin-drying times, (C) ventilation rates, and (D) numbers of compression cycles were varied. There were no significant differences between the values (*p* > 0.05). The data are means with their standard deviations (*n* = 3).

SSO manufactured under various processing conditions showed stable oxidative characteristics, in terms of their acid values (Fig 5) or iodine values (Fig 6). The acid and iodine values are important attributes for the quality of SSO. Edible oils are mainly composed of lipids, which are vulnerable to oxidation, and so the KMFDS regulates such oxidative stabilities to within certain limits. According to the Korean Food Code (KMFDS, 2015) [37], SSO should have acid value of less than 4.0 and iodine value of 103–118. As shown in Fig 5, although the means of the acid values varied somewhat under different processing conditions, the differences were not statistically significant regarding their standard deviations, and all values were under the limit of 4.0. It has been reported that the acid values of vegetable oils are stable for up to 5 months [38], and since SSO has a higher oxidative stability compared to other vegetable oils due to the presence of lignans and tocopherols [39,40], the acid values of SSO investigated in this study could be considered to be stable during storage. The iodine values also showed nonsignificant variations, but some values were slightly lower than the mandated range limits of 103–118 because of the prolonged storage duration [38,41]. However, the degree of variations were similar to the study reported by Abou-Gharbia et al. [41], which was proven to be nonsignigicant (*p* < 0.05). Thus, the invented apparatus ensures an oxidative stability while reducing the BaP content in SSO.

### Total phenolic content

**Fig 7:**
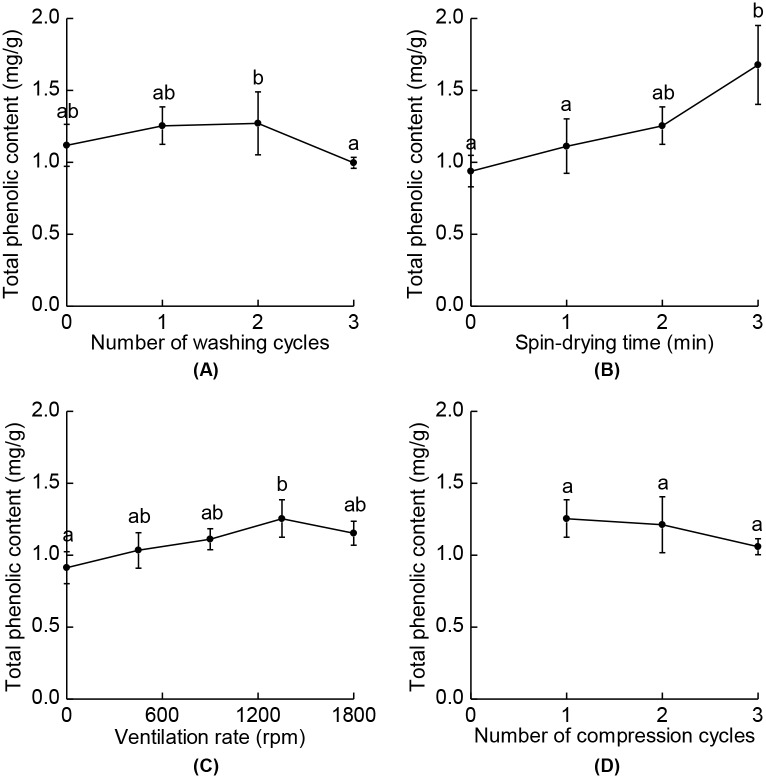
Total phenolic contents of SSO for different processing conditions. (A) Numbers of washing cycles, (B) spin-drying times, (C) ventilation rates, and (D) numbers of compression cycles were varied. Different letters indicate significant differences between the values (*p* < 0.05). The data are means with their standard deviations (*n* = 3).

Higher total phenolic content was presented in SSO with lower BaP contents, not only supporting a good oxidative stability of SSO mentioned above but also showing the possible effect of antioxidants on reducing BaP contents. Phenolic compounds which are reported to be abundant in SSO are known as important antioxidants and they can form stable radical intermediates by donating a hydrogen atom or an electron [30,42]. As shown in Fig 7, total phenolic contents of SSO were measured in the range of 0.91–1.68 mg/g, which are reasonable regarding previous studies [30,43]. Higher total phenolic contents were obtained in SSO for no, one, and two cycles of washing, for 2–3 min of spin-drying time, and for 450–1800 rpm of ventilation rate. These were also shown to be optimal conditions for reducing BaP contents. Thus, it could be suggested that some of phenolic antioxidants presented in SSO had possibly reduced BaP contents during the manufacture of SSO, as previous studies has reported that the addition of antioxidants lead to the reduction in BaP contents of cooking oil fumes [44] or inactivation of BaP metabolites [45].

### Sesamol and sesamolin content

Sesamol contents in SSO for different processing conditions presented similar patterns to the results obtained for total phenolic content, and these were reversal patterns to those of BaP content (Tables 1–4). Since it has been reported that the composition of antioxidants in SSO varied with processing conditions during thermal oxidation [13,41], we assumed that the composition of antioxidative lignans was changed with varying conditions during the roasting process and this have led to the differences in total phenolic content. Table 1 indicates that sesamol contents decreased and sesamolin contents increased as the number of washing cycle increased. This could be due to elevated moisture content of SS that causes elongated roasting time, since a continuous heat supply induces decomposition of sesamolin to produce sesamol [46]. Sesamol contents also increased with spin-drying time, but sesamolin contents remained statistically constant (*p* > 0.05). In this case, another feature of lignans can be applied: decomposition of sesamol is promoted with moisture of SS during thermal processing [46]. As mentioned above, both sesamol and sesamolin have high oxidative stability and show BaP inactivation effect. However, sesamol has been reported to show stronger antioxidant capacity than other free radical scavengers at high temperature [47], and it can also inhibit autoxidation of lipids [48]. Thus, sesamol could probably prevent BaP formation in SSO that involves free radical reactions, intramolecular cyclization, and polymerization [22,23]. Unlike the results of Tables 1 and 2, the effect of ventilation rate on sesamol and sesamolin contents (Table 3) did not show reversal patterns to the results of BaP content, and this was because of another factor, a gas removing effect. Modeling of roasting for studying the emission rate of roasting gases and the corresponding thermal distribution of the roasting machine would further elaborate BaP lowering effect of the invented device, by determining the optimal point that maximizes the ventilation effect while maintain a uniform heat distribution to prevent BaP formation. The number of compression cycle affected sesamol content (Table 4) in SSO, but it also did not show such a reversal pattern to the BaP content result. In fact, controlling the number of compression cycle did not change BaP content, since the operation temperature was lower compared to the roasting step and its time is not long enough so that it cannot produce additional BaP.

**Table 1:** Sesamol and sesamolin contents in SSO for different numbers of washing cycles.

Washing cycle	Sesamol content (mg/l)	Sesamolin content (mg/l)
0	81.97 ± 3.99 b	100.98 ± 16.18 a
1	77.00 ± 11.23 b	102.32 ± 7.03 a
2	80.03 ± 10.89 b	104.34 ± 11.80 a
3	49.33 ± 11.76 a	131.61 ± 3.47 b

Different letters in the same column indicate significant differences between the values (*p* < 0.05). The data are means with their standard deviations (*n* = 3).

**Table 2:** Sesamol and sesamolin contents in SSO for different spin-drying times.

Spin-drying time (min)	Sesamol content (mg/l)	Sesamolin content (mg/l)
0	42.32 ± 3.69 a	106.59 ± 6.85
1	64.81 ± 11.03 b	101.97 ± 22.81
2	77.00 ± 11.23 b	102.32 ± 7.03
3	69.14 ± 6.66 b	104.23 ± 15.84

Different letters in the same column indicate significant differences between the values (*p* < 0.05). The data are means with their standard deviations (*n* = 3).

**Table 3:** Sesamol and sesamolin contents in SSO for different ventilation rates.

Ventilation rate (rpm)	Sesamol content (mg/l)	Sesamolin content (mg/l)
0	93.74 ± 17.67 ab	103.85 ± 4.83
450	112.17 ± 28.93 b	88.71 ± 25.66
900	94.03 ± 2.09 ab	105.09 ± 4.08
1350	77.00 ± 11.23 a	102.32 ± 7.03
1800	78.40 ± 13.62 a	103.41 ± 9.24

Different letters in the same column indicate significant differences between the values (*p* < 0.05). The data are means with their standard deviations (*n* = 3).

**Table 4:** Sesamol and sesamolin contents in SSO for different numbers of compression cycles.

Compression cycle	Sesamol content (mg/l)	Sesamolin content (mg/l)
1	77.00 ± 11.23 b	102.32 ± 7.03
2	78.66 ± 8.66 b	109.18 ± 14.30
3	34.87 ± 3.46 a	121.98 ± 11.00

Different letters in the same column indicate significant differences between the values (*p* < 0.05). The data are means with their standard deviations (*n* = 3).

### Comparison of the BaP content in oils manufactured by traditional and self-designed apparatuses

We were able to lower the BaP content in SSO to 0.20 μg/kg with the self-designed apparatus, while the traditional one resulted in the BaP content of 0.30 μg/kg. Since we applied the optimal processing conditions, the range of the BaP content in SSO was low, but it was still showed the lower value with the self-designed apparatus. Moreover, this trend was consistent with perilla oil, which is a sister product of SSO. The BaP content in perilla oil of the self-designed apparatus was 0.60 μg/kg, where that of the traditional one was 0.80 μg/kg.

## Conclusions

The BaP content in the final oil products can be successfully reduced by the self-designed apparatus. The optimal processing conditions of the apparatus involve a single cycle of washing, 2 min of spin-drying, 1350-rpm ventilation during roasting, and a single cycle of compression, which in the present study reduced the initial contamination of 10-μg/kg BaP to 2.93 μg/kg. In addition to improved safety, the antioxidative capacities were verified as being uniform throughout the experiments, showing acceptable acid and iodine values for commercial SSO products. The inclusion of an automatic control system ensures such uniform qualities, in contrast to the variations that characterize manually controlled conventional systems that were identified in preliminary tests. Moreover, SSO with lower BaP contents presented higher total phenolic contents and it was suggested that sesamol might have prevented BaP formation due to its antioxidative capacity.

In further investigations of BaP content reduction mechanism, the apparatus invented in this study could serve as a standard model for the commercial manufacture of low-BaP SSO. Macroscale modeling of roasting for studying the emission rate of roasting gases and the corresponding thermal distribution of the roasting machine would enhance the BaP content reduction by maximizing the ventilation effect while maintaining a uniform heat distribution with minimum heat loss. In addition to quantitative analyses of antioxidants, chemical analyses of the compositions of fatty acids, proteins in SSO manufactured under different conditions would clarify the reduction mechanism and improve the understanding of this new technology.
